# Proteomic analysis of knee cartilage reveals potential signaling pathways in pathological mechanism of Kashin-Beck disease compared with osteoarthritis

**DOI:** 10.1038/s41598-020-63932-6

**Published:** 2020-04-22

**Authors:** Jian Lei, Abebe Feyissa Amhare, Liyun Wang, Yizhen Lv, Huan Deng, Hang Gao, Xiong Guo, Jing Han, Mikko J. Lammi

**Affiliations:** 1 School of Public Health, Health Science Center; Key Laboratory of Environment and Gene Related Diseases of Ministry Education; Key Laboratory of Trace Elements and Endemic Diseases, Ministry of Health, Xi’an Jiaotong University, Xi’an, Shaanxi 710061 P. R. China; 2 Shenzhen Institute, Xi’an Jiaotong University, Shenzhen, Guangdong 518057 P. R. China; 3 Laboratory of Resource Biology and Biotechnology in Western China (Ministry of Education), Provincial Key Laboratory of Biotechnology, College of Life Sciences, Northwest University, Xi’an, Shaanxi 710069 P. R. China; 4 Department of Integrative Medical Biology, Umeå University, Umeå, 90187 Sweden

**Keywords:** Osteoarthritis, Cartilage

## Abstract

The pathological mechanism of Kashin-Beck disease (KBD), an endemic osteoarthritic disease, remains to be poorly understood. This study was designed to identify signaling pathways and crucial proteins involved in the pathological mechanism of KBD compared with osteoarthritis (OA). The knee cartilage samples were collected from gender- and age-matched KBD (n = 9) and OA (n = 9) patients. After pre-processing, samples were labeled with Tamdem Mass Tags 6plex multiplex kit, and analyzed by liquid chromatography-tandem mass spectrometry. Proteomic results were analyzed with gene ontology (GO), Kyoto Encyclopedia of Genes and Genomes (KEGG), and protein-protein interactions (PPI). The differential abundance proteins from KBD and OA were validated using western blot analysis. As a result, A total number of 375 proteins were identified to have differential abundance between KBD and OA, of which 121 and 254 proteins were observed to be up-regulated or down-regulated in KBD group. GO analysis shows that the differential abundant proteins are associated with cell junction and signal transducer activity from extracellular to intracellular. KEGG pathways enrichment and PPI network indicate four major pathways, including extracellular matrix -receptor interaction, focal adhesion, phosphatidylinositol 3-kinase (PI3K)-Protein kinase B (Akt), and Ras signaling pathways were involved in the degeneration of cartilage. Moreover, integrins, laminins, NF-κB and other regulative molecules were found as crucial proteins. In conclusion, our results demonstrated that compared with OA, the differential abundance proteins and signaling pathways may contribute to the occurrence and development of joint damage in KBD. Further investigation of their regulative roles and interaction may provide new insights into the pathological mechanisms and therapeutic targets for KBD.

## Introduction

Osteoarthritis (OA) is a progressive, degenerative joint disease. It is the major cause of knee pain and locomotor disability worldwide^1,2^. The WHO Scientific Groupon Rheumatic Diseases estimates that 10% of the world’s population who are 60 years or older have significant clinical problems attributed to OA^3^. Unlike OA, Kashin-Beck disease (KBD) is a chronic and serious endemic osteoarticular disease, which has been in high prevalence and morbidity in Eastern Siberia of Russia, Northeast China to Sichuan-Tibet Plateau, and North Korea^4,5^. According to the statistics, there were 1.04 billion people in risk of KBD and 0.57 million patients with KBD in China^6^. However, the etiology and pathological mechanisms of KBD are still waiting to be uncovered^7^, which lessen the chances of further effective prevention and treatment measures for the current KBD patients.

Several similar pathological changes have been found in KBD and OA, including degradation of extracellular matrix (ECM), cartilage lesion, reduction and disruption of proteoglycans (PGs) and collagens^5,8–10^. While the causes and pathological mechanism of the KBD and OA differ in many aspects. OA occurs mainly in the middle aged and elderly population^1^. The pathologic changes in OA joints include degradation of the articular cartilage, especially at the superficial zones, thickening of the subchondral bone, osteophyte formation, and variable degrees of synovial inflammation^11^. KBD occurs mostly in children aged 3–12 years old in endemic regions^4^. Most of the deformities were established at the age of 15, then subsequently become symptomatic in adults^7,12,13^. A typical distinction of KBD from OA or other bone and joint diseases is the damage of epiphyseal cartilage and hyaline cartilage in the deep zones of cartilage^11,14^. This distinction might explain the occurrence of severe joint deformities during development in young people with KBD^15^.

After taking some efficient efforts in the past decades, including changing the source of drinking water, using selenium supplements, exchanging grains from non-endemic areas and other controlling measures, the prevalence of KBD has been in control^4,9^. The incidence of KBD is rare nowadays, and only 2.24% of the current patients are under13 age^6^. The local farmers and the immigrating populations are the major population suffering from KBD^4^. KBD caused pain and deformities in joints and ability lost in doing farm work, which increased economic burdens to their family and society^3–5^. Meanwhile, the current adult KBD patients are mostly advanced KBD often accompanied by OA^8,10^. The lack of systematical proteomic analysis of KBD and OA cause theoretical blankness of pathological mechanism research on KBD.

This study was designed to identify the proteins in differential abundance between KBD and OA, and enrich the signaling pathways the proteins involved by using bioinformatics analysis. It is expected to reveal the initial signal transduction procession and contribute to the pathological mechanism investigation of KBD.

## Methods

### Sample collection

For the proteomic analysis, 18 knee arthritic cartilage samples were collected. 9 KBD samples were obtained from the patients between 54–68 years of age, who were living in the KBD-endemic areas of Linyou in Shannxi province. 9 OA samples were obtained from non-KBD-endemic areas from Xi’an in Shaanxi province (Detailed subjects characteristics are shown in Table 1). The KBD and OA cartilage donors were age- and gender-matched Chinese Han lineage. OA was diagnosed based on the Western Ontario and McMaster Universities OA Index^16^. KBD was diagnosed as grade I or grade II based on the clinical criteria used for the diagnosis of KBD (WST 207–2010) in China^12,13,16^. The damage degrees of OA and KBD cartilage were diagnosed as grade 2–3.5 based on histopathology staining^17^. Articular cartilage tissues were collected from the same anatomical position of the femoral condyles. This investigation was approved by the Human Ethics Committees of Xi’an Jiaotong University. All procedures performed in this study involving human participants were in accordance with the 1964 Helsinki declaration and its later amendments or comparable ethical standards. All the donors were provided a written informed consent for the study participation and publication of their individual clinical details and images.

**Table 1:** Characteristics of experiment subjects (χ2 gender = 0.222, Fage = 1.650, both p value > 0.05).

KBD			OA		
Sample Set	Age	Gender	Sample Set	Age	Gender
1	50	F	1	51	M
2	58	F	2	56	F
3	50	M	3	68	M
4	55	M	4	58	M
5	61	M	5	60	F
6	66	F	6	57	F
7	54	F	7	67	F
8	68	M	8	55	M
9	67	F	9	61	M
Mean	58.778	—	Mean	59.222	—

OA: Osteoarthritis; KBD: Kashin-Beck Disease; Control: Normal adult; F: Female; M: Male.

### Sample preparation

After collection, the articular cartilage tissues were cut, snap-frozen for 5 minutes using liquid nitrogen and stored at −80 °C. For each group, 3 samples were mixed together as 1 biological replicate. then homogenized by grinding and weighted in precooled 2 ml conical tubes, and 0.8 ml solution containing 4% SDS, 100 mM Tris-HCl, 100 mM 1,4-dithiothreitol, pH 8.0 was added (10%, v/w). The mixtures were boiled and homogenized by ultrasonication in ice bath and centrifuged. The supernatant was filtered with 0.22 µm ultrafiltration device. 1 µl of each sample was quantitated with BCA kit (SK3021, Sangon Biotech, Shanghai, China), then the samples were aliquoted in tubes to contain 20 μg protein each, and stored at −80 °C.

### TMT labeling and high-pH chromatography separation

The samples were weighted at 300 μg for filter aided sample preparation enzymolysis, and this procession was strictly in accordance with the protocol of universal sample preparation method for proteome analysis^18^. The filtrates were collected, and the peptides of the samples were quantitated by measuring optical density at 280 nm. 100 μg of the samples were labeled with Tandem Mass Tag (TMT) 6plex Isobaric Label Reagent Set (Thermo Scientific, Rockford, IL, USA). In both KBD and OA groups, 9 samples have been pooled into 3 samples, each with 3 patients, and 6 samples was acquired finally. The total 6 mixed samples were subjected to High-pH Reversed-Phase Fractionation in 1100 Series HPLC Value System (Agilent Technologies, Waldbronn, Germany) equipped with a Gemini-NX column (00F-4453-E0, 4.6 × 150 mm, 3 μm, 110 Å, Phenomenex, Torrance, CA, USA), which was eluted at a flow rate of 0.8 ml/minute. The whole elution process was monitored by measuring the absorbance at 214 nm, and fractions were collected every 1.25 minutes. Finally, the collected fractions (approximately 40) were combined into ten fractions. Each fraction was concentrated by vacuum centrifugation and reconstituted in 40 μl of 0.1% v/v trifluoroacetic acid. The samples collected were stored at −80 °C.

### Quantitative LC-MS/MS Analysis

Quantitative proteomic based on liquid chromatography-tandem mass spectrometry (LC-MS/MS) was applied to proteins collected from the former procedure. LC-MS/MS analysis was performed on an Easy nLC nanoflow HPLC system connected to Thermo Scientific Orbitrap Fusion Tribrid mass spectrometer (Thermo Fisher Scientific, San Jose, CA, USA). A total of 1 μg samples were loaded with an auto-sampler onto a Thermo Scientific EASY column (2 columns, at a flow rate of 150 nl/minute), then separated in the Thermo Scientific EASY trap column (100 µm * 2 cm, 5 µm, 100 Å, C18) and analytical column (75 μm×25 µm, 5 µm, 100 Å, C18) with a segmented 2 hours gradient of solvent A (0.1% formic acid in water) to 35% of solvent B (0.1% formic acid in 84% acetonitrile) for 100 minutes, followed by 35 – 90% of solvent B for 3 minutes, and 90% of solvent B for 5 minutes. The column was re-equilibrated to its initial highly aqueous solvent composition before each analysis.

The mass spectrometer was operated in positive ion mode, and MS spectra were acquired over a range of 375–1500 m/z. The resolving powers of the MS scan and MS/MS scan at 200 m/z for the Fusion were set as 120,000 and 50,000, respectively. Data Dependent Mode was Top Speed, Cycle Time 3 seconds and ions were fragmented through higher energy collisional dissociation. The maximum ion injection times were set to 50 ms for the survey scan and 105 ms for the MS/MS scans, and the automatic gain control target values for Master scan modes were set to 4e5, and for MS/MS 1e5. The dynamic exclusion duration was 40 seconds.

### Mass spectrometry and Bioinformatics analysis

Proteome Discoverer 2.1 software (Thermo Fisher Scientific) was used to analyze the raw files. (Matrix Science) embedded in Proteome Discoverer was used to search the raw data against the uniprot_human_156914_20170308.fasta (Total 156914 sequences). Peptide mass tolerances of ± 20 ppm and fragments of mass tolerance of 0.1 Da were used for parent and monoisotopic fragment ions. All data were reported based on 99% confidence for protein identification, as determined by ≤0.01 (High Confident) false discovery rate (FDR) using a decoy database. Peptides and proteins with FDR less than 0.01 were accepted and further processed. The relative quantitative analysis of the proteins in the samples based on the ratios of TMT reporter ions from all unique peptides. The relative peak intensities of the TMT reporter ions released in the MS/MS spectra were used for quantification. Then, the final ratios obtained from the relative protein quantifications were normalized based on the median average protein quantification ratio.

Principal component analysis (PCA) has been conducted to investigate the homogeneity within groups and heterogeneity between groups. Proteins in statistically differences and the changing ratio over 1.2 were further analyzed for functional and biological relevance. Differentially expressed proteins expressed with a log form were input as hierarchical clustering algorithms for gene ontology (GO) analysis derived from the UniProt-gene ontology annotation (GOA) database. The identified differentially expressed proteins were annotated as three categories, i.e. biological process, cellular component, and molecular function, based on GO enrichment analysis. Pathway enrichment analysis of differential expressed proteins was performed using KEGG database. Enriched GO terms were identified with Fisher’s Exact Test.

In addition, the identified proteins were sent to the String database (https://string-db.org/) to predict and visualize the protein-protein interactions (PPI) among the differentially expressed proteins. The represented network has been obtained at a medium confidence in data settings with min interaction score as 0.4.

### Validation of protein expressions

For the validation of the differentially expressed proteins, western blotting was used for quantitative analysis. Cartilage tissues were lysed by 2% sodium dodecyl sulfate with 2 M urea, 10% glycerol, 10 mM Tris-HCl (pH 6.8), 10 mM dithiothreitol and 1 mM phenylmethylsulfonyl fluoride, then the lysates were centrifuged. The supernatants were separated by SDS-PAGE and blotted onto a nitrocellulose membrane (Bio-Rad Laboratories, Hercules, CA, USA). After blocking with 5% skimmed milk, the membrane was then analyzed using specific antibodies and visualized by enhanced chemiluminescence (ECL Kit, Amersham Biosciences, Little Chalfont, UK). The primary antibodies to Indian hedgehog (IHH, ab52919), type I collagen (COL1A1, ab138492), type II collagen (COL2A1, ab188570), aggrecan (ACAN, ab3778) and glyceraldehyde-3-phosphate dehydrogenase (GAPDH, ab9894) were obtained from Abcam (Cambridge, UK). Protein signals were detected using secondary HRP-conjugated anti-mouse or anti-rabbit antibodies by the enhanced chemiluminescence (ECL Kit, Amersham Biosciences, Little Chalfont, UK). The full-length images of individual blots were displayed in Fig. S1.

## Results

### Proteomic results

Considering the complex biological differences in human samples, PCA results showed the homogeneity and heterogeneity in and between KBD and OA groups (Fig. S2). The proteomics results can identify differential expressed protein between the two groups. In the result, a total of 11362 unique peptides corresponding to 2205 proteins were identified (Table S1). Protein intensity based absolute quantification values were used to describe the protein abundance. Proteins with an abundance difference ratio ≥ 1.20 and *p* <0.05 (t-test) were considered as up-regulated proteins, and those with ratio ≤0.83 as down-regulated proteins. Following this criterion, a total of 375 proteins (121 proteins up- and 254 down-regulated in KBD group) were defined as differentially expressed proteins in comparison with OA group (Table S2).

### GO terms and GO enrichment analysis

To understand the functions and biological processes of the differentially abundant proteins involved in the KBD group, we performed GO term and GO enrichment analysis with GO enrichment software. Multiple corrections for GO has been conducted and showed with q value.

**Figure 1:**
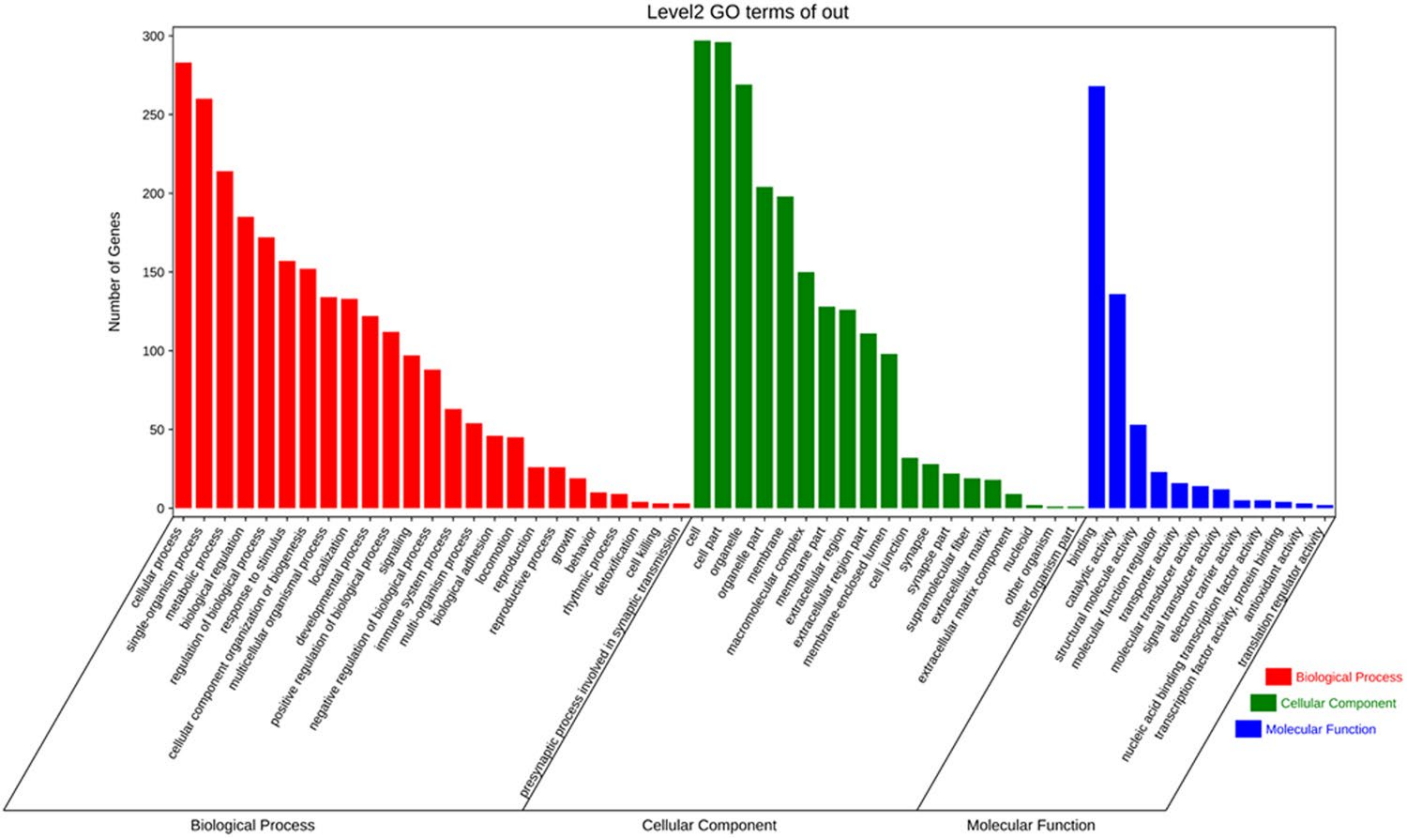
GO term analysis of proteins differentially expressed in KBD compared with OA.

GO terms results showed that the top abundant terms are: binding, structural molecular activity, molecular transducer activity, and signal transducer activity (molecular function); macromolecular complex, membrane part, extracellular region, and cell junction (cellular component); cellular procession, metabolic process, localization, and response to stimulus (biological process) (Fig. 1). This result indicates that the differential metabolic activity in KBD could be associated with cell junction and signal transducer activity from extracellular region to intracellular.

**Figure 2:**
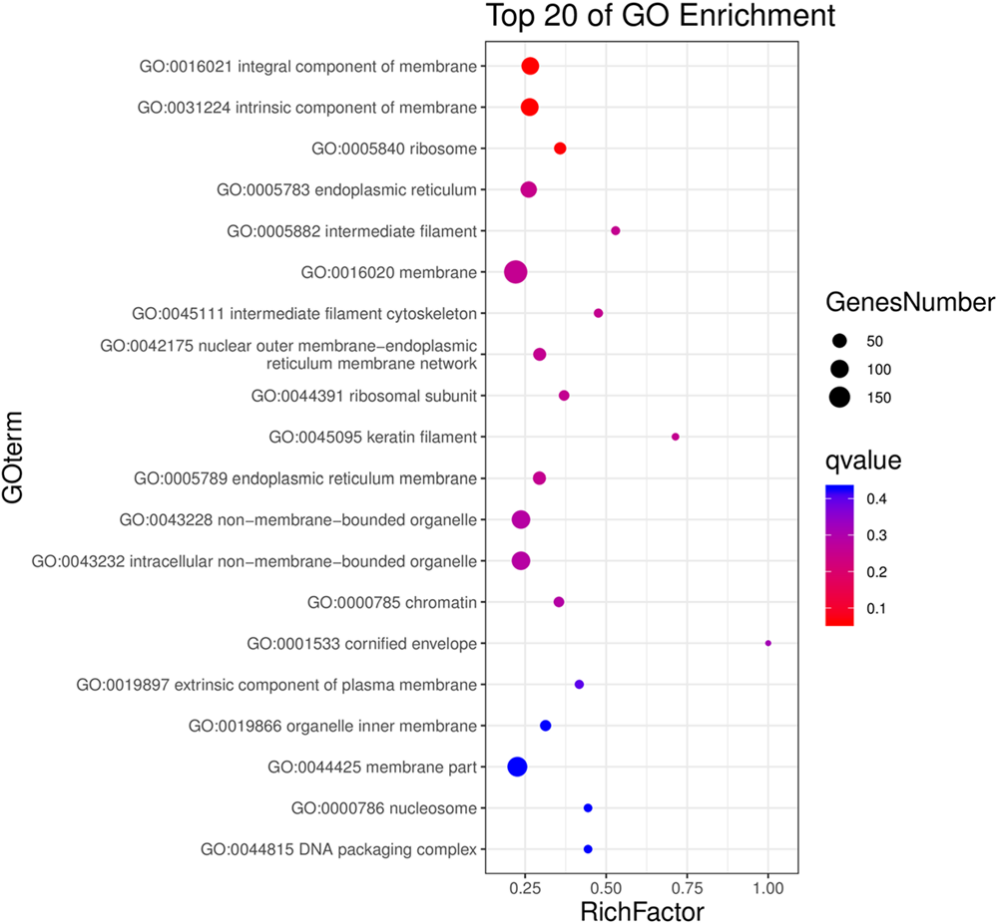
Top 20 GO enrichment analysis results of proteins differentially expressed in KBD compared with OA.

The GO enrichment analysis shows that integral component of membrane, intrinsic component of membrane, ribosome, endoplasmic reticulum, intermediate filament, membrane, intermediate filament cytoskeleton, are the major GO terms of altered proteins (Fig. 2). The results of GO enrichment and GO term analysis are in high consistence, and the identified biological processes can be marked as entry points for the further pathological mechanism investigation.

### KEGG enrichment analysis

To investigate the functions and signaling pathways, which the differentially expressed proteins were involved in, KEGG automatic annotation server (KAAS) was used to search the identified proteins in the KEGG GENES database. KEGG enrichment analysis and multiple corrections were conducted and showed with q value.

**Figure 3:**
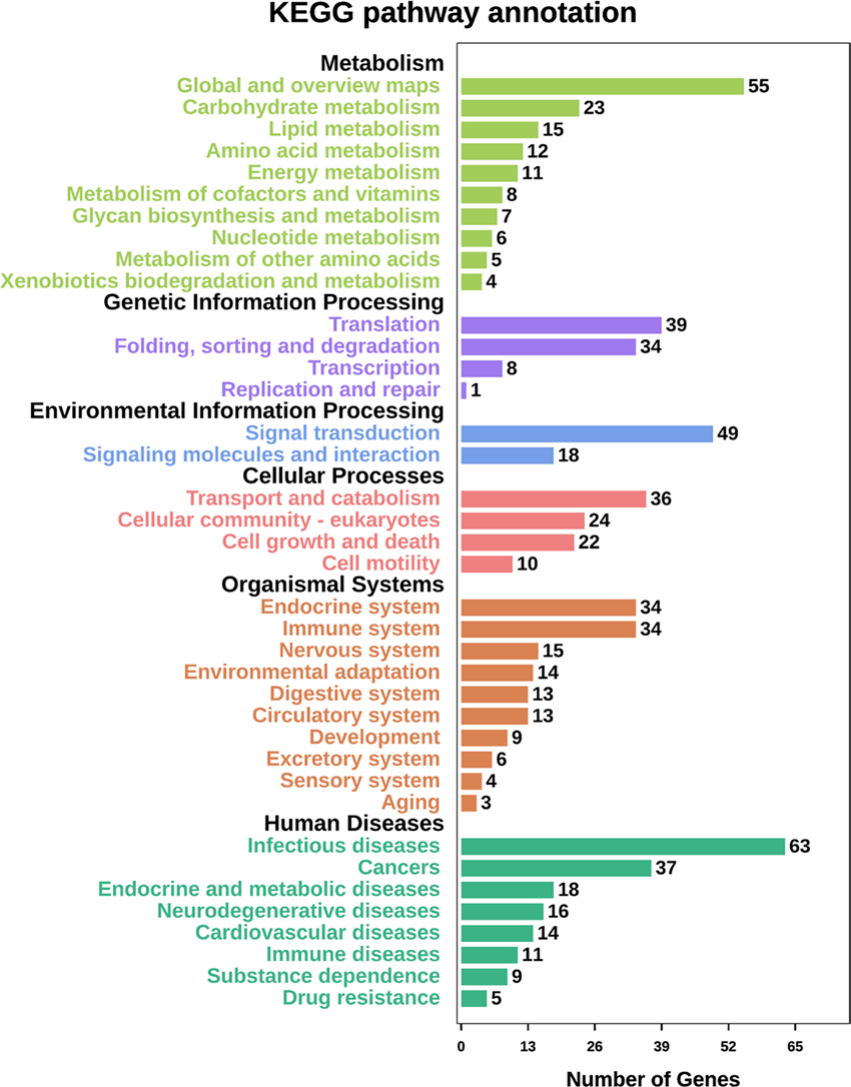
KEGG annotation analysis results of proteins differentially expressed in KBD compared with OA.

**Figure 4:**
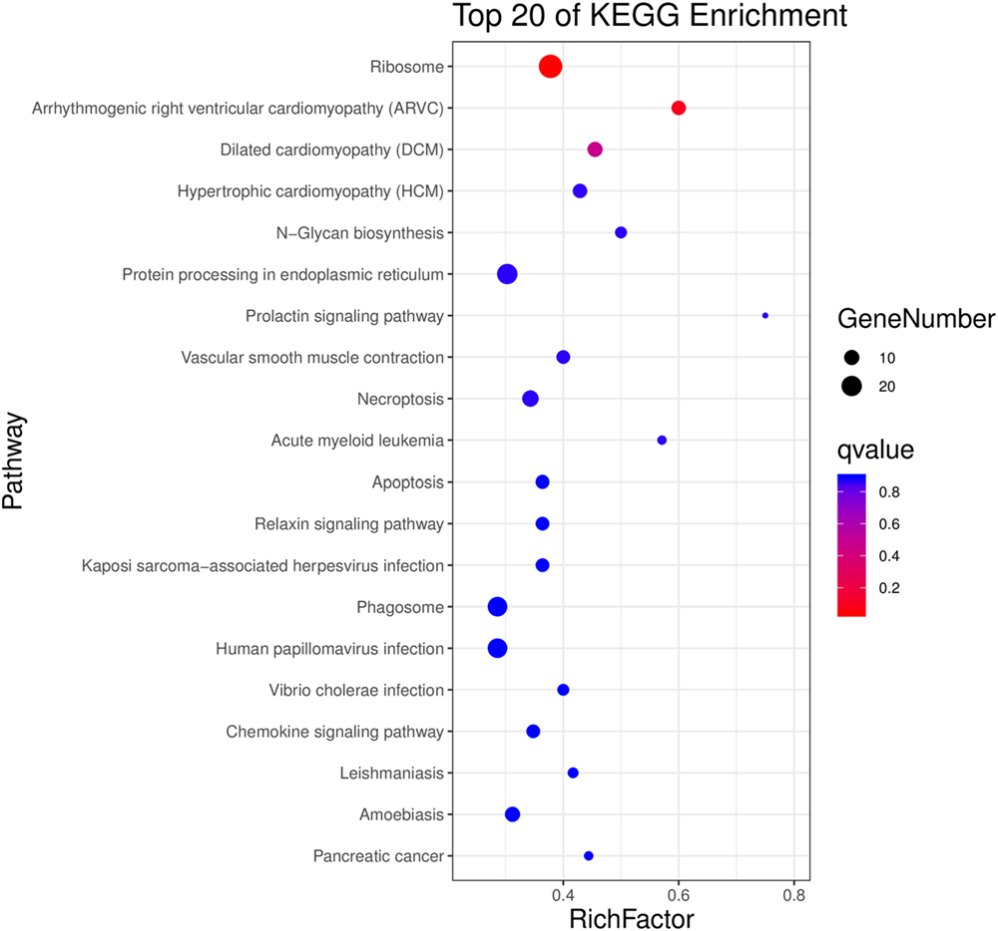
Top 20 KEGG enrichment analysis results of proteins differentially expressed in KBD compared with OA.

As shown in KEGG annotation, major metabolisms are carbohydrate metabolism, lipid metabolism; major genetic information processing are translation, folding, sorting and degeneration; major environmental information processing are signal transduction, signaling molecules and interaction; major cellular processes are transport and catabolism, cellular community-eukaryotes; organismal systems are endocrine systems, immune system; the detailed information was showed in Fig. 3. And the results of KEGG enrichment were showed in Fig. 4.

**Figure 5:**
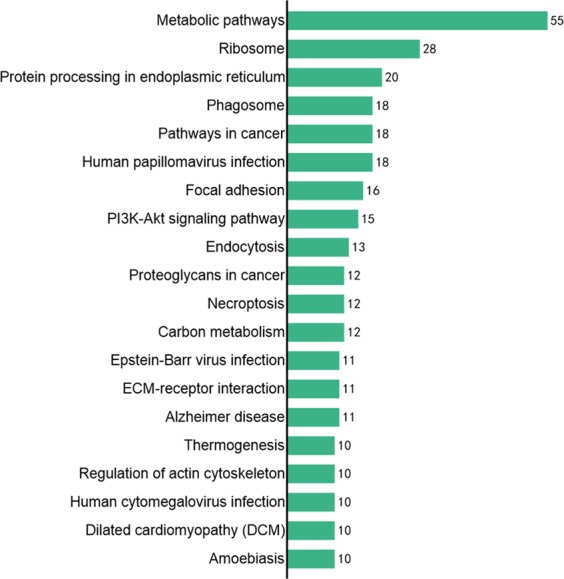
Top 20 signaling pathways ranked by the number of involved proteins differentially expressed in KBD compared with OA.

Total of 254 differentially expressed proteins were mapped in 245 signaling or metabolic KEGG pathways. Then the signaling pathways were ranked by the number of proteins involved. Focal adhesion, phosphatidylinositol 3-kinase (PI3K)-Akt signaling pathway, ECM-receptor interaction signaling pathway were identified in the proteins differentially expressed in KBD (Fig. 5). These signaling pathways are closely related to chondrocyte metabolisms, such as cell junction activity and signaling transducer activity from extracellular region to intracellular.

In addition, key proteins MAPK3, RELA, STAT3, ROCK1, ITGB3, ITGA1, LAMA2, COL4A1, COL1A2, COL1A1, THBS3, THBS4, RALA, GNG5, GNB1, and GNG5 were revealed to be closely related to chondrocyte metabolism (Table 2). These results implied potential targets for further research of signaling pathways involved in KBD, which might provide necessary clues for the investigation of the pathological mechanism of KBD.

**Table 2:** The TOP relative proteins ranked by the involved signaling pathway numbers.

Uniprot Entry ID	KBD vs. OA	Gene	Protein	Pathway name
L7RXH5	down	MAPK3	**Mitogen-activated protein kinase**	Cellular senescence; Autophagy; Insulin signaling pathway; Regulation of actin cytoskeleton; Phospholipase D signaling pathway; Estrogen signaling pathway; Osteoclast differentiation; MAPK signaling pathway; Toll-like receptor signaling pathway; Th1 and Th2 cell differentiation; AGE-RAGE signaling pathway in diabetic complications; Adherens junction; IL-17 signaling pathway; Proteoglycans in cancer; Chemokine signaling pathway; TNF signaling pathway; Signaling pathways regulating pluripotency of stem cells; VEGF signaling pathway; GnRH signaling pathway; Apoptosis; Focal adhesion; TGF-beta signaling pathway; NOD-like receptor signaling pathway; PI3K-Akt signaling pathway; Ras signaling pathway; Rap1 signaling pathway; cGMP-PKG signaling pathway; mTOR signaling pathway; cAMP signaling pathway; T cell receptor signaling pathway;
Q04206	up	RELA	**Transcription factor p65**	Cellular senescence; Mitophagy; Osteoclast differentiation; Fluid shear stress and atherosclerosis; MAPK signaling pathway; Toll-like receptor signaling pathway; Th1 and Th2 cell differentiation; AGE-RAGE signaling pathway in diabetic complications; NF-kappa B signaling pathway; IL-17 signaling pathway; Chemokine signaling pathway; TNF signaling pathway; Apoptosis; NOD-like receptor signaling pathway; RIG-I-like receptor signaling pathway; PI3K-Akt signaling pathway; Ras signaling pathway; HIF-1 signaling pathway; cAMP signaling pathway; Th17 cell differentiation; T cell receptor signaling pathway;
A0A1W6S962	down	GTF2I	**GTF2I-BRAF fusion protein**	Insulin signaling pathway; Regulation of actin cytoskeleton; MAPK signaling pathway; Proteoglycans in cancer; Vascular smooth muscle contraction; Natural killer cell mediated cytotoxicity; Focal adhesion; Rap1 signaling pathway; mTOR signaling pathway; cAMP signaling pathway;
P40763	down	STAT3	**Signal transducer and activator of transcription 3**	AGE-RAGE signaling pathway in diabetic complications; Proteoglycans in cancer; Signaling pathways regulating pluripotency of stem cells; HIF-1 signaling pathway; Jak-STAT signaling pathway; Th17 cell differentiation;
D9ZGF8	down	ROCK1	**Rho-associated protein kinase**	Regulation of actin cytoskeleton; Proteoglycans in cancer; Focal adhesion; TGF-beta signaling pathway; cGMP-PKG signaling pathway; cAMP signaling pathway; Tight junction;
L7UUZ7	down	ITGB3	**Integrin beta**	Regulation of actin cytoskeleton; Phagosome; Proteoglycans in cancer; ECM-receptor interaction; Focal adhesion; PI3K-Akt signaling pathway; Rap1 signaling pathway;
P63218	down	GNG5	**Guanine nucleotide-binding protein G(I)/G(S)/G(O) subunit gamma-5**	Chemokine signaling pathway; PI3K-Akt signaling pathway; Ras signaling pathway; GABAergic synapse;
P02462	down	COL4A1	**Collagen alpha-1(IV) chain**	AGE-RAGE signaling pathway in diabetic complications; ECM-receptor interaction; Focal adhesion; PI3K-Akt signaling pathway;
P56199	down	ITGA1	**Integrin alpha-1**	Regulation of actin cytoskeleton; ECM-receptor interaction; Focal adhesion; PI3K-Akt signaling pathway;
P08123	down	COL1A2	**Collagen alpha-2(I) chain**	AGE-RAGE signaling pathway in diabetic complications; ECM-receptor interaction; Focal adhesion; PI3K-Akt signaling pathway;
P02452	down	COL1A1	**Collagen alpha-1(I) chain**	AGE-RAGE signaling pathway in diabetic complications; ECM-receptor interaction; Focal adhesion; PI3K-Akt signaling pathway;
P11233	down	RALA	**Ras-related protein Ral-A**	Ras signaling pathway; Rap1 signaling pathway;
P24043	up	LAMA2	**Laminin subunit alpha-2**	ECM-receptor interaction; Focal adhesion; PI3K-Akt signaling pathway;
Q15904	up	ATP6AP1	**V-type proton ATPase subunit S1**	Rheumatoid arthritis; Oxidative phosphorylation; Metabolic pathways; Phagosome; Lysosome;
P08138	up	NGFR	**Tumor necrosis factor receptor superfamily member 16**	MAPK signaling pathway; PI3K-Akt signaling pathway; Ras signaling pathway; Rap1 signaling pathway; Apoptosis - multiple species;
P49746	up	THBS3	**Thrombospondin-3**	Phagosome; ECM-receptor interaction; Focal adhesion; PI3K-Akt signaling pathway;
P35443	up	THBS4	**Thrombospondin-4**	Phagosome; ECM-receptor interaction; Focal adhesion; PI4K-Akt signaling pathway;

### Protein-protein interaction network

**Figure 6:**
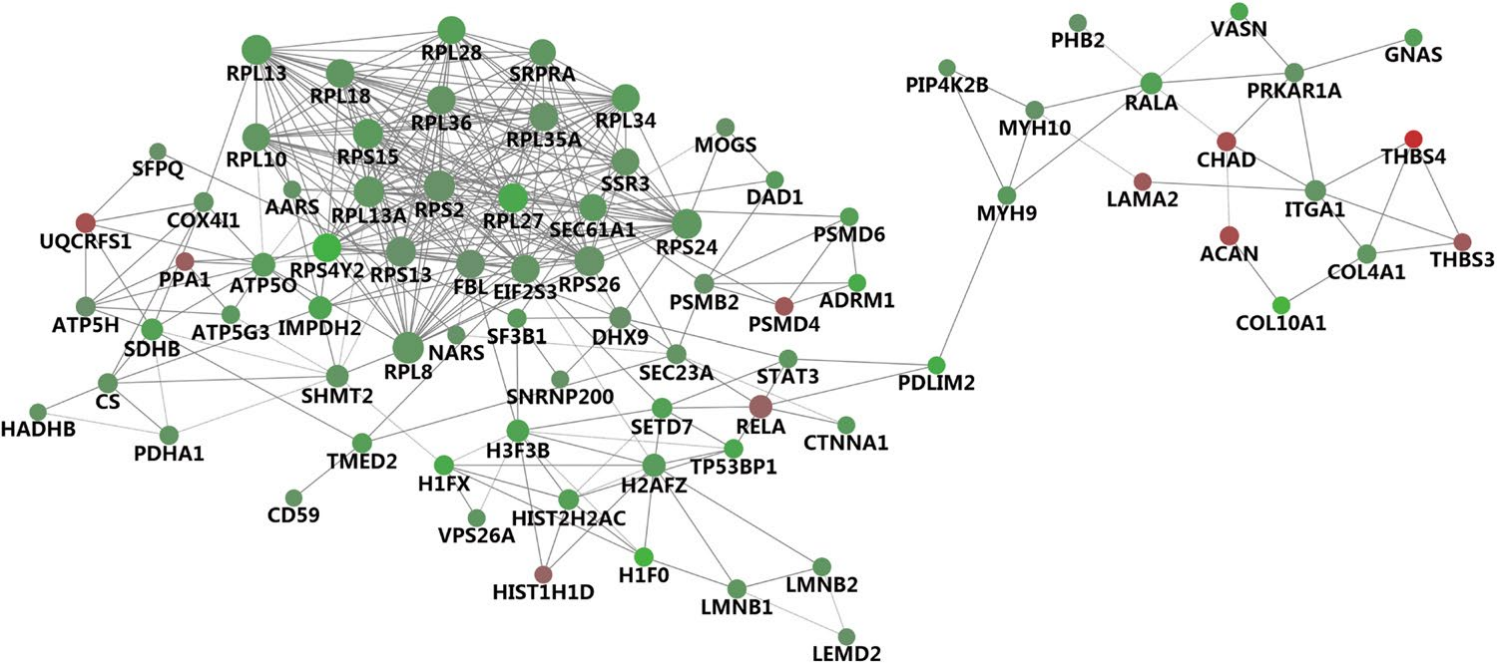
Protein interaction network analysis of proteins differentially expressed in KBD compared with OA. The relationship of proteins differentially expressed in KBD compared with OA group. “─” means “direct relationship”; red indicates up-regulated proteins, green indicates down-regulated proteins. Confidence: 0.4.

Different sorts of proteins interact with each other and participate in a series of biochemical reactions in order to perform their biological functions. To obtain more clues of the functions of the abundant proteins differentially expressed, protein-protein interaction networks were conducted with STRING and Cytoscape databases. As the result, the central role of proteins like Ras-related protein Ral-A (RALA), integrin alpha-1 (ITGA1), transcription factor p65 (NF-κB, RELA), signal transducer and activator of transcription 3 (STAT3), cAMP-dependent protein kinase type I-alpha regulatory subunit (PRKAR1A), collagen alpha-1(IV) chain (COL4A1) appeared crucial in the regulation of differential expression of proteins (Fig. 6).

### Western blot validation

**Figure 7:**
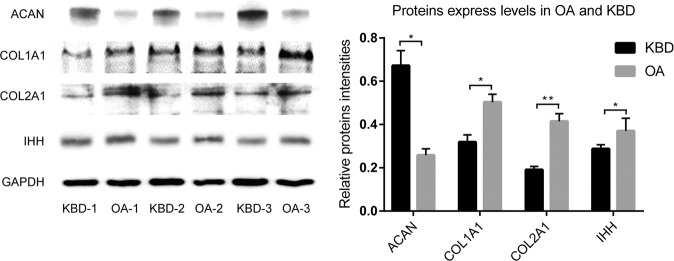
Verification experiments of key proteins expression in KBD group compared with OA. Representative images of the blots are shown in the lift panels, and the results of the semiquantitative analysis performed by their densitometric analysis are shown at the right. * p < 0.05, ** p < 0.01.

To validate the alterations in expression of these proteins in KBD and OA group, equal amounts of protein samples were analyzed by Western blot with specific antibodies. The expression level of IHH (hedgehog subfamily), COL2A1, and COL1A1 was decreased in KBD compared with OA, while aggrecan (ACAN) was increased in KBD (Fig. 7). Thus, it can be seen that the protein expression trends are in high consistency with proteomic assay results.

## Discussion

TMT labeling combined with high-pH chromatography separation and quantitative LC-MS/MS analysis is an efficient, high-throughput, and accurate technology method to analyze the expression levels, locations, functions, and interactions of proteins. More proteins can be identified with TMT labeling proteomics than 2-D DIGE proteomics. Previous study has conducted proteomics analysis between KBD and health group with 2-D DIGE proteomics^14^. Previous study and our study both identified down regulated phosphoglycerate kinases (PGKs) in KBD. PGK1 was identified in the previous study and PGK2 in our study. Furthermore, in our results, four central pathways (ECM-receptor interaction, focal adhesion, PI3K-Akt signaling pathway, Ras signaling pathway) were identified in the proteins differentially expressed in KBD. These results may provide potential signaling pathways for further investigation of KBD pathological mechanism.

Articular cartilage ECM consists of a complex mixture of PGs, collagens, aggrecan, and serves an important role in the maintenance of tissue morphogenesis and function^19^. Former studies observed decreased content of PGs and aggrecan in the deep zone of KBD cartilage^20–23^. In this study, the protein-protein interaction and KEGG pathways analyses indicated that collagens (COL1A1, COL1A2, and COL4A1), were relatively decreased in the KBD group. Besides, certain other ECM components, such as aggrecan (ACAN), CHAD, LAMA2, THBS3, THBS4, and transmembrane ECM receptor protein integrin (ITGA1, ITGB3) are in differential abundance in KBD group.

Integrin is a family of transmembrane proteins, which can bind ECM macromolecules and the specificity depending on the pairing of one α- and one β-chain^24^. The interacting of integrin and ECM molecules participate in many signaling pathways of the chondrocytes, and function in cell survival, growth, differentiation and tissue remodeling. Integrin-mediated cell adhesion is known to be crucial for mechanical and structural support of ECM^25,26^. The disruption of integrin-mediated cell-matrix interactions has remarkable effects on cartilage metabolism and maintenance of cartilage homeostasis^24^. Deficiency of α_2_β_1_ integrin (collagen receptor) was associated with lowered severity of arthritic symptoms in mouse model of osteoarthritic disease^27^. As a member of collagen receptor dimer, the relative down-regulation of ITGA1 is in line with the expressions of COL1A1, COL1A2, and COL4A1. Previous studies have indicated that integrins can transduce signals from the ECM into the intracellular processes, and participate in regulation of cell differentiation, matrix remodeling, responses to stimulation, and cell survival^26,28^. The molecular functions of differentially expressed integrin in the pathological mechanism of KBD would warrant further studies.

Focal adhesion is a key ECM-receptor interaction signaling pathway, which integrins also participate in. The ECM binding to integrins signals cells to phosphorylate focal adhesion kinase (FAK), and integrin/FAK interaction has been shown to mediate mechanochemical transduction in chondrocytes^29^. Focal adhesion distribution was known to be important in osteogenic, adipogenic and chondrogenic guidance^30^. Dynamics of actin cytoskeleton is dependent on Rho-associated coiled-coil containing protein kinases 1 and 2 (ROCK1 and ROCK2). And Rho/ROCK pathway was reported to regulate the expression of chondrocyte-specific genes Sox9, ACAN and COL2A1^31,32^. Laminins are a family of heterotrimeric ECM proteins^33^. Previous studies have shown the expressions of specific laminins were differentially regulated during development, adhesion, migration and regeneration^34–36^. In our study, LAMA2 was observed to be up-regulated in KBD group in comparison to OA. These proteins were involved in ECM-receptor interaction signaling pathway, which might suggest the regulative functions in chondrocyte related gene expression.

Our proteomic results showed the expression of NF-κB, GNG5, GNB1, and other guanine nucleotide-binding (G) proteins in Ras and PI3K-Akt signaling pathways altered in KBD. The G-proteins are participated in transmitting extracellular signals to intracellular signal pathways, and evidence indicated G-proteins can affect the pathophysiology of arthritic diseases^37^. NF-κB is a vital protein in cartilage metabolism. NF-κB-regulated gene products are involved in inflammation and cartilage degradation^11,38^. Moreover, NF-κB pathway enhances the articular damage by promoting the synthesis of catabolic factors, cartilage inflammation and apoptosis of chondrocyte^39,40^. RALA is an important protein in chondrocytes, it was reported that the knockdown of RALA during chondrogenesis was noticed to remarkably increase Sox9 protein^41^. Former research indicated that bone morphogenetic proteins (BMPs) can stimulate both chondrocyte ECM synthesis and chondrocyte differentiation^42^. Besides, BMPs can prevent apoptosis in chondrocytes via PI3K/AKT-mediated activation of NF-κB^43^.

The proteomic results also identified several proteins and signal pathways worthy of investigation, such as MAPK, Jak-STAT signaling pathways, phagosomes, ribosomes, and metabolic pathways. It has been reported that PI3K, PKC, and p38-MAPK pathways can regulate the chondrogenic phenotype^44^. The activation of MAPK signaling pathway was critical during embryonic cartilage and joint development^45^, and research also indicated that both MAPK and Wnt family participate in chondrogenesis and cartilage development^42^, which is consistent with our results that several proteins involved in Wnt signaling pathway were in differential expression. The inhibition of STAT is known to transactivate the expression of alkaline phosphatase and type X collagen^46,47^, and our results also indicated that the NF-κB protein involved in Jak-STAT signaling pathway was up-regulated, and STAT protein down-regulated, which can influence the process of chondrogenesis^48^. In general, signaling pathways involved in KBD have important regulative roles in the pathogenesis and progression of KBD, and the cellular metabolism are compromised by the production of antianabolic, pro-catabolic and pro-inflammatory factors^49^.

In conclusion, this research identified the key signal pathways from with proteomics, which were connected by the key proteins, such as LAMA2, integrins, NF-κB, and G proteins. Although the roles of the signaling pathways have not been completely understood, we speculate that these signaling pathways are involved in the pathological mechanisms of KBD. The PPI results showed the key information nodes of different signaling pathway networks, which reflected the intracellular behavior of gene and protein expression stimulated by extracellular mechanical or biochemical information. Thus, the key proteins or pathways are potential targets for pathological mechanisms and therapies. Further investigations are expected to provide new insights into the molecular regulation between ECM and chondrocytes, and contribute to the pathological mechanisms of KBD.

## Supplementary information

Supplementary Information.

Supplementary Information2.

Supplementary Information3.
