# Supplementary material for: Proteomic analysis of knee cartilage reveals potential signaling pathways in pathological mechanism of Kashin-Beck disease compared with osteoarthritis
Source: Sci Rep. 2020 Apr 22;10:6824. doi: 10.1038/s41598-020-63932-6 (PMC7176695; doi:10.1038/s41598-020-63932-6)
Supplement: Supplementary file 1 — Supplementary Information. [file 41598_2020_63932_MOESM1_ESM.pdf]

Proteomic analysis of knee cartilage reveals potential signaling pathways in pathological mechanism of Kashin-Beck disease compared with osteoarthritis

Jian Lei, Amhare Abebe Feyissa, Liyun Wang, Yizhen Lv, Huan Deng, Hang Gao, Xiong Guo, Jing Han*, Mikko J. Lammi

Figure S1 Full-length blots of ACAN, COL1A1, COL2A1, IHH, GAPDH.

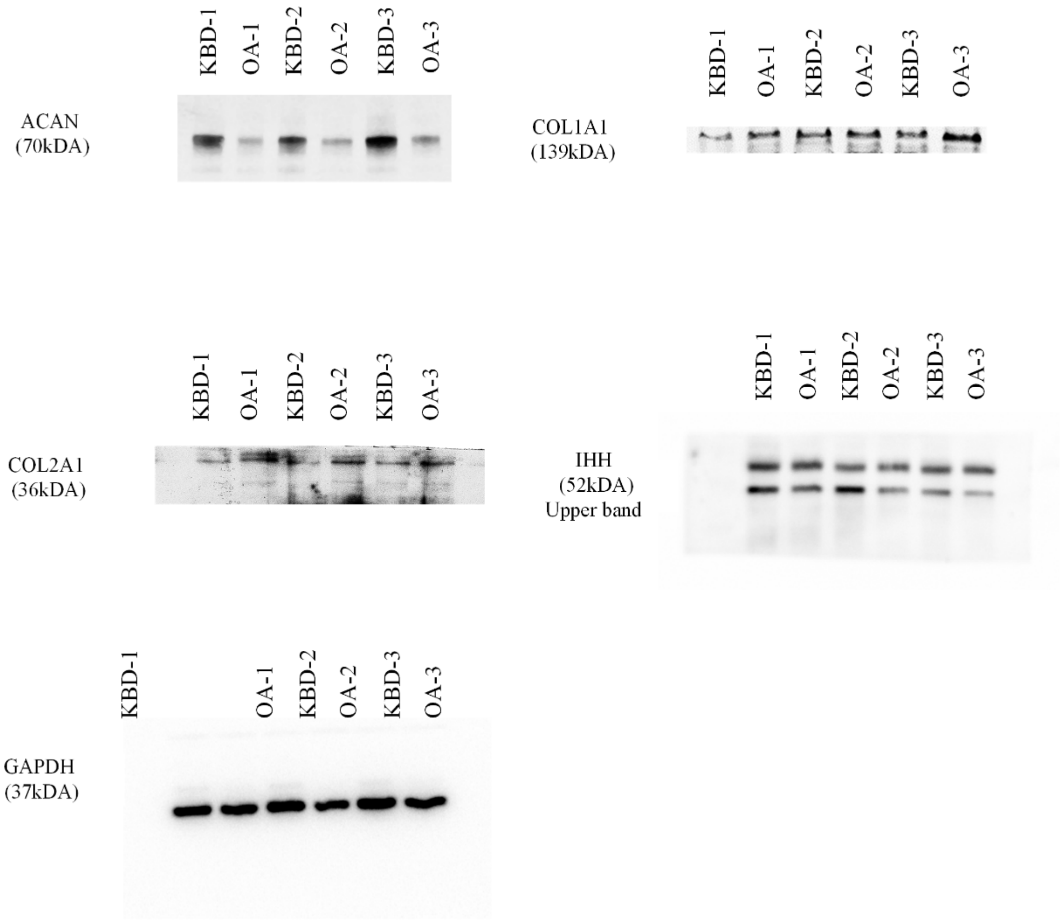

Figure S2 PCA 2D scores plots of mixed cartilage samples in KBD and OA

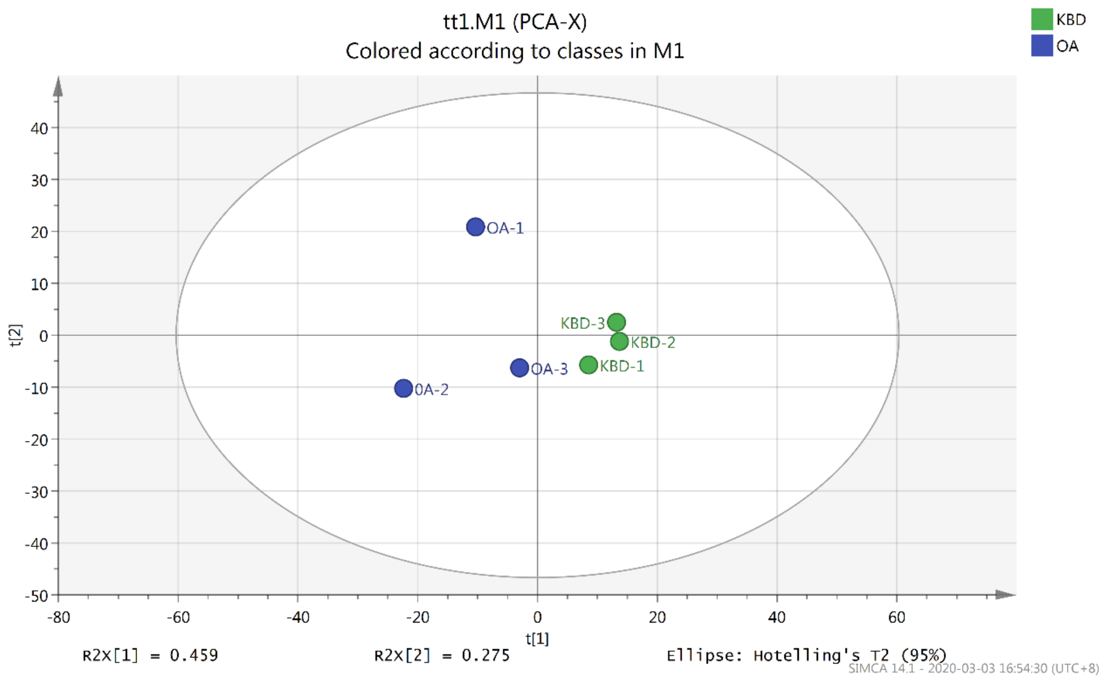
tt1.M1 (PCA-X)
Colored according to classes in M1
KBD
OA
OA-1
KBD-3
KBD-2
KBD-1
OA-3
OA-2
t[2]
t[1]
R2X[1] = 0.459
R2X[2] = 0.275
Ellipse: Hotelling's T2 (95%)
SIMCA 14.1 - 2020-03-03 16:54:30 (UTC+8)
